# Developmental expression of immune-associated secreted novel AID/APOBEC-like deaminases (SNAD1s) in common carp *(Cyprinus carpio)*

**DOI:** 10.3389/fimmu.2026.1893787

**Published:** 2026-07-13

**Authors:** Anna Małgorzata Majewska, Alexander Rebl, Mariola Dietrich, Tomáš Korytář, Jiří Kyslík, Veronika Piačková, Roman Franěk, Andrzej Ciereszko, Mikołaj Adamek

**Affiliations:** 1 Gamete Biology Team, InLife Institute of Animal Reproduction and Food Research, Polish Academy of Sciences, Olsztyn, Poland; 2 Research Institute for Farm Animal Biology (FBN), Working Group Fish Genetics, Dummerstorf, Germany; 3 Laboratory of Fish Immunology, Institute of Parasitology Biology Centre, Czech Academy of Sciences, Ceske Budejovice, Czechia; 4 Faculty of Fisheries and Protection of Waters, South Bohemian Research Center of Aquaculture and Biodiversity of Hydrocenoses, University of South Bohemia, České Budějovice, Czechia; 5 Fish Disease Research Unit, Institute for Parasitology, University of Veterinary Medicine, Hannover, Germany

**Keywords:** common carp, early larval development, embryogenesis, immunology, nucleic acids editing, secreted cytidine deaminases 1 (*snad1s*)

## Abstract

Early developmental stages of teleost fish represent vulnerable phases of the life cycle, characterized by exposure to fluctuating abiotic and biotic environmental conditions. Immune system maturation is gradual, beginning with reliance on maternally transferred immune factors, followed by activation of the innate immune system and culminating in the establishment of adaptive immunity. Recent studies in common carp (*Cyprinus carpio*) have identified a novel family of cytidine deaminases termed Secreted Novel AID/APOBEC-like Deaminases (SNAD1s). Although their biological functions remain unknown, preliminary evidence suggests that *snad1* genes may participate in immune- and stress-related processes. In the present study, we analyzed the expression of 13 *snad1* genes during embryonic and early larval development of common carp. Twelve of the thirteen genes (excluding *snad_506*) exhibited significant stage-dependent variation in expression and formed four distinct temporal expression patterns. Four paralogous genes (*snad_318*, *snad_800*, *snad_835*, and *snad_810*) were maternally expressed, suggesting deposition in the oocyte and potential involvement in early developmental regulation. In contrast, *snad_409*, *snad_208*, and *snad_962* displayed delayed post-zygotic activation, with increased expression observed during later developmental stages coinciding with post-hatching maturation of immune-related physiological processes and establishment of host defense mechanisms. Comparative analysis of publicly available RNA-seq datasets confirmed conserved *snad1* expression dynamics across independent studies and methodological approaches. This study provides the first comprehensive overview of developmental *snad1* gene expression during vertebrate ontogeny. The dynamic and stage-dependent expression of *snad1* genes reveals distinct developmental regulation patterns and highlights substantial heterogeneity within the carp *snad1* gene family during ontogeny.

## 1 Introduction

Early developmental stages of teleost fish are highly susceptible to fluctuating environmental conditions and microbial exposure (1, 2). Following hatching, embryos and larvae are directly exposed to abiotic stressors, including water temperature and chemistry, as well as to diverse microbial communities and fish pathogens (2, 3). These challenges may be further exacerbated under climate change-related conditions (4), contributing to high and often unpredictable mortality during early ontogeny in both natural populations and aquaculture systems (5, 6). Consequently, efficient larval production remains one of the major bottlenecks in modern aquaculture.

Protection during embryonic and larval development initially relies on maternally transferred immune factors, including immunoglobulins and innate effector molecules, whereas autonomous immune competence is established progressively during ontogeny (5, 7, 8). In addition to maternal transcripts encoding protective molecules such as complement components and antimicrobial peptides (AMPs), developing larvae acquire the capacity to express immune-related genes shortly after fertilization (9). This process coincides with the maternal-to-zygotic transition (MZT), during which the zygotic genome becomes transcriptionally active and initiates the expression of genes involved predominantly in innate immune responses; in zebrafish, zygotic genome activation begins approximately 3 h post-fertilization (10). In common carp (*Cyprinus carpio*), adaptive immune functions are not fully established until 1–2 months after fertilization (11). Therefore, understanding the ontogenetic development of immune mechanisms is important both for elucidating fundamental aspects of fish immunology and for improving protection of vulnerable early life stages in aquaculture (12).

Activation-induced cytidine deaminase/apolipoprotein B mRNA editing catalytic polypeptide-like deaminases (AID/APOBECs; AADs) are zinc-dependent deaminases that catalyze the deamination of bases in nucleic acids, resulting in cytidine to uracil conversion (13). This leads to alterations in the genetic information carried by nucleic acids, resulting in a broad range of genomic and epigenetic modifications that modulate both innate and adaptive immune responses (14). Phylogenetic sequences analysis published in 2018 revealed the presence of Secreted Novel AID/APOBEC-like deaminases (SNAD1s), distinguished by the presence of a signal peptide, which are unique compared to classical intracellular AID/APOBECs (13, 15). *Snad1* genes are present, among vertebrates, only in lower poikilothermic lower vertebrates, including fish. Our laboratory obtained the first evidence for the presence of SNAD_810 protein in fish, which was identified in carp blood plasma during cold acclimation (16).

Although the biological functions of SNAD1 proteins remain largely unknown, accumulating evidence suggests their involvement in immune and stress responses (17). In our recent study, we characterized the phylogeny and structural diversity of the SNAD1 family and reannotated several previously uncharacterized fish proteins and transcripts as SNAD1 homologues (17). Comparative analyses demonstrated broad conservation of SNAD1 sequences among fish species and revealed pronounced modulation of their expression during innate and adaptive immune responses, cold-enhanced immunity, and stress-related processes (17). Importantly, data from zebrafish larvae support a conserved role of *snad1* genes during early vertebrate development (17).

Common carp (*Cyprinus carpio*) is one of the most important freshwater aquaculture species worldwide and an established vertebrate model complementary to zebrafish (18). Owing to their external development and continuous exposure to microbe-rich aquatic environments, carp embryos and larvae rely heavily on innate defense mechanisms during early ontogeny. In light of the emerging evidence linking SNAD1 proteins to immune-related processes in fish (17), understanding their developmental regulation may provide important insights into the molecular basis of early host defense. Notably, our recent analyses identified 13 *snad1* genes in the carp genome, highlighting an unexpected expansion of this family and the need for a comprehensive assessment of their expression dynamics during development (17). The unusually large number of *snad1* paralogs in common carp may be linked to the complex evolutionary history of cyprinid genomes, including the additional whole-genome duplication that occurred in the Cyprinus lineage, although the precise mechanisms underlying snad1 family expansion remain unresolved.

Therefore, the aim of the present study was to characterize the expression profiles of all 13 currently identified *snad1* genes throughout common carp embryogenesis. Using multiplex PCR, transcript abundance was analyzed at key developmental stages ranging from fertilization to hatching (0, 17, 26, 49, 74, 98, 123, 147, 170, 190, and 240 hours post fertilization (hpf). The results revealed distinct temporal expression patterns among *snad1* family members, suggesting differential regulation during early ontogeny and a potential contribution to the establishment of immune competence. To our knowledge, this study provides the first comprehensive analysis of *snad1* gene expression during vertebrate development and establishes a framework for future functional investigations of this novel family of secreted cytidine deaminases.

## 2 Materials and methods

Unless otherwise stated, all reagents were purchased from Sigma-Aldrich (St. Louis, MO, USA).

### 2.1 Ethics statement

All experimental protocols and methods involving live fish in this study were approved by the Ethics Committee of the University of South Bohemia in České Budějovice, Faculty of Fisheries and Protection of Waters. European Union Directive 2010/63/EU and Czech national legislation (Act no. 246/1992 Coll., Section 29, as amended by Act no. 70/2025 Coll.).

### 2.2 Broodstock maintenance for the induced breeding

BV mirror carp breed fish weighing ∼ 6.5 kg were maintained in the broodstock rearing fishpond of the University of South Bohemia in České Budějovice, Faculty of the Fisheries and Protection of Waters, South Bohemian Research Center of Aquaculture and Biodiversity of Hydrocenoses in Vodňany, Czechia. During the growing season (April – September), the broodstock fed on natural food (zooplankton, zoobenthos, periphyton etc) and a supplemental feed (wheat grains). During winter (October – March) no supplemental feeding was applied, water quality (temperature, oxygen saturation, pH) was monitored once a week and, if necessary, the holes were cut in ice cover to enable gas exchange between water and air. In March the broodstock was harvested and transferred to a manipulation stock pond for pre-spawning sorting.

### 2.3 Assessment of the gonadal maturation and pre-spawning preparation

Fish were previously individually tagged with P.I.Ts. Fish were selected for later spawning according to their records (age, previous reproduction data, if any) and the visual assessment of the fish health status and visual and palpation examination of the fish pre-spawning preparation status. Males were selected for spawning in case of good health condition and spermiation after gentle massage of the belly. Females were selected for good health status and based on the shape, size and hardness of their belly. Selected fish were kept in shallow fishponds of 0.16 ha, separated by sex, and fed on natural food and plant-based carp pellets (KP2, Výroba krmiv spol. s r.o.- Krmné směsi Stříbrné Hory, Czechia) until their harvest and transfer to a fish hatchery in May.

### 2.4 Induced spawning and egg incubation of the mirror common carp

Fish were placed in the fish hatchery in indoor rubber-textile basins of 4.5 m^3^ separated by sex and kept at water temperature of 18 °C until next day. Males were injected with a minced carp pituitary extract (CPE) in physiological saline at dose 1 mg CPE× kg^-1^ ∼ 24 hours prior to spawning; females were injected with two doses of CPE ∼ 24 hours (0.5 mg CPE× kg^-1^) and 12 hours (2.5 mg CPE× kg^-1^) prior to spawning. After CPE injection of fish, the water temperature was gradually increased to 21 °C. Sperm was collected into 250 mL tissue-culture containers, eggs were collected in dry plastic dishes of 1 L. Eggs of 5 females (50 mL of eggs per female) were pooled together, inseminated with sperm of 5 males (1 mL of sperm per male) using heterospermy and fertilized by adding of 250 mL of hatchery water (21 °C) upon gentle stirring. Egg stickiness was eliminated 10 min. post fertilization using tannic acid (TA) solution (2 g of TA × L^-1^ of hatchery water) for 5 min. Afterwards, the eggs were incubated in 9L Zugar jars at 21 °C.

### 2.5 Studies on the embryonic developmental process – sampling procedures

Fertilized eggs were allowed to adhere to glass Petri dishes (~50 per 9-cm dish) and maintained in a recirculating system with aeration and UV-treated water at 21 °C. Prior to each imaging timepoint, a subset of developing embryos was incubated in 0.1% pronase for 10 min under gentle agitation. After chorion softening, embryos were manually dechorionated using fine forceps and kept in 1x Ringer solution. Imaging was conducted using a Leica FA205 stereomicroscope equipped with a Leica DMC6200 camera and LAS X acquisition software. Sampling points were selected to represent major developmental and morphological transitions during common carp ontogeny. Samples were collected at the following stages: 0 hpf, 17 hpf, 26 hpf, 49 hpf, 74 hpf, 98 hpf, 123 hpf, 147 hpf, 170 hpf, 190 hpf, and 240 hpf (Summary of embryonic development data; **Supplementary Table 1**). For each developmental stage, four independent biological samples were collected from the pooled progeny obtained from the same fertilization event. Each biological replicate was processed independently for RNA extraction and subsequent gene-expression analyses.

Gene expression profiling focused on all thirteen SNAD1 genes (*snad_835, snad_160, snad_063, snad_769, snad_962, snad_946 snad_448, snad_318, snad_800, snad_409, snad_208, snad_810, snad_506*).

### 2.6 RNA isolation, primer design, and multiplex quantitative PCR

Gene expression analysis comprised a total of 13 target genes. Oligonucleotide primers specific to *Cyprinus carpio* (**Supplementary Table 2**) were designed using the Pyrosequencing Assay Design software v1.0.6 (Biotage). Whenever possible, either the forward or reverse primer was designed to span an exon–exon junction to prevent genomic DNA amplification. In addition, primers were selected to enable the detection of multiple transcript variants or paralogs in cases where several gene isoforms were present. All primers were validated prior to multiplex qPCR analysis using the LightCycler 96 system (Roche). Reactions were optimized for a final volume of 12 µl, comprising 6 µl SensiFAST SYBR No-ROX Mix (Bioline/Meridian Bioscience), 5 µl cDNA, and 1 µl primer mix (10 µM). The LightCycler protocol consisted of an initial denaturation at 95 °C for 5 min, followed by 40 cycles of 95 °C for 5 s, 60 °C for 15 s, and 72 °C for 15 s. Fluorescence was measured at 72 °C for 10 s during each cycle. Melting curve analyses confirmed amplification specificity and the absence of non-specific products. Amplicon sizes ranging from 74 to 210 bp were verified by electrophoresis on 2% agarose gels containing ethidium bromide and visualized using a ChemiDoc MP imaging system (Bio-Rad).

Total RNA was extracted from whole embryos/larvae using the ISOLATE II RNA Mini Kit (Bioline/Meridian Bioscience). Residual genomic DNA was removed by digestion with RNase-free DNase I (Qiagen) for 12 min at 37 °C. RNA concentration and purity were assessed using a NanoDrop OneC spectrophotometer (NanoDrop Technologies).

Gene-expression profiling was conducted using the integrated fluidic circuit (IFC) technology of Fluidigm Gene Expression biochips. Multiplex analyses were performed on ten 48 Dynamic Array IFC chips (Standard BioTools) with the BioMark HD System (Standard BioTools). For cDNA synthesis, 1 µl of total RNA was reverse-transcribed using the Reverse Transcription Master Mix (Standard BioTools) under the following conditions: 25 °C for 5 min, 42 °C for 30 min, and 85 °C for 5 min. The resulting cDNA was adjusted to 10 ng/µl, combined with primers (100 µM) and PreAmp Master Mix (Standard BioTools), and preamplified for 14 cycles (95 °C for 15 s; 60 °C for 4 min) in a TAdvanced thermocycler (Biometra). After preamplification, exonuclease I (New England BioLabs) was added to remove single-stranded primers, followed by dilution with 43 µl TE buffer (Sigma-Aldrich/Merck) per sample. Each 50 µl cDNA sample was subsequently diluted in SsoFast EvaGreen Supermix with Low ROX (Bio-Rad) and 20× DNA Binding Dye Sample Loading Reagent (Standard BioTools) to prepare the sample mixes. After priming the 48.48 IFC chips in the MX Controller (Standard BioTools), the primer and sample mixes were introduced into the designated inlets. Multiplex qPCR was performed using the manufacturer’s protocol “GE Fast 48×48 PCR+Melt v2.pcl.” Data were extracted using the Fluidigm Real-Time PCR Analysis Software v4.8.1 and normalized using global mean normalization, which involves scaling the expression of each target gene relative to the average expression of all genes measured within the same sample.

Preliminary evaluation of the candidate reference genes actb, ef1α, and 40S revealed developmental stage-dependent variation in transcript abundance (data not shown). Therefore, global mean normalization was selected as the most appropriate normalization strategy for samples undergoing extensive transcriptional remodeling during embryogenesis.

### 2.7 Statistical analysis

Statistical analysis was performed by StatSoft Poland (www.StatSoft.pl) using Statistica software.

One-way ANOVA was used to evaluate differences between sampling times. Normality of data was tested using Shapiro-Wilk test and equality of variance using Brown-Forsythe test. Depending of latter, F Welch or F test for one-way Anova were used, together with Tukey’s *post-hoc* test.

A hierarchical cluster analysis was conducted to evaluate the similarity of the mean profiles of the analyzed variables. The analysis was performed using Ward’s linkage method with Euclidean distance as the measure of dissimilarity. This approach minimized the total within-cluster variance and allowed for the identification of relatively homogeneous groups of observations. The number of clusters was determined by visual inspection of the dendrogram. A linkage distance threshold of 7.5 was selected based on the dendrogram structure and agglomeration schedule, resulting in six clusters composed of genes exhibiting highly similar developmental expression profiles.

## 3 Results

### 3.1 Snad1s expression dynamics during carp ontogeny

**Figure 1:**
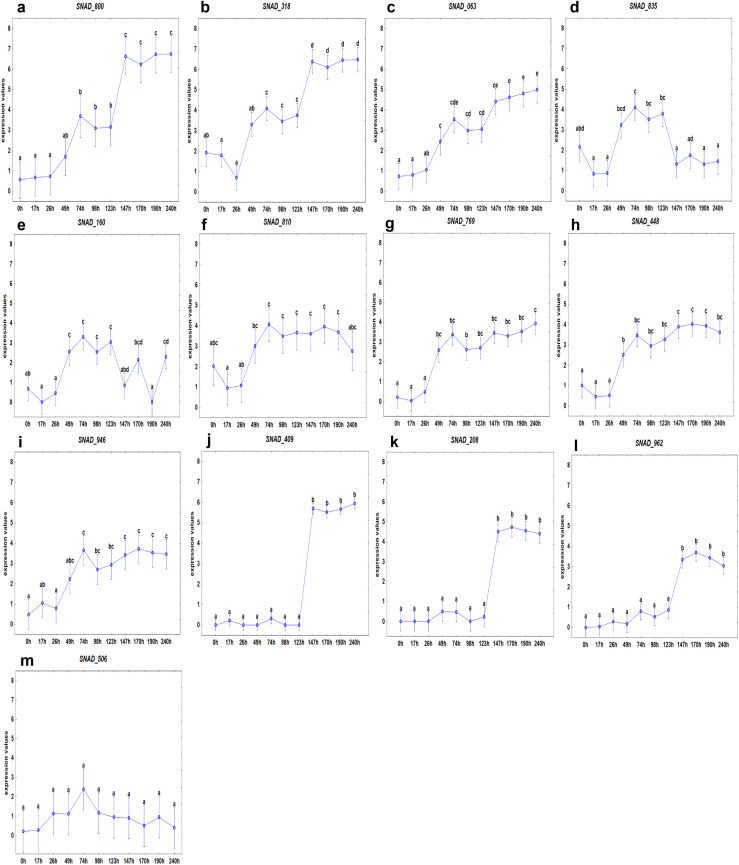
Temporal changes in expression of 13 *snad1* family genes across developmental stages of common carp. Panels **(a–m)** represent individual genes. Significant differences are indicated by different letters (Tukey’s HSD test, α = 0.05).

For 12 of the 13 *snad1* genes (excluding *snad_506*), expression differed significantly across developmental stages (**Figures 1A–M**).

*Snad_800* and *snad_318* showed a marked increase in expression between 26 and 74 hpf, followed by a second pronounced upregulation starting at 123 hpf, reaching peak expression at 147 hpf and remaining elevated thereafter (**Figures 1A, B**). *Snad_063* displayed similar pattern, although the secondary increase after 123 hpf was less pronounced (**Figure 1C**). A comparable early increase in expression between 26 and 74 hpf was also detected for *snad_835* and *snad_160* (**Figures 1D, E**). In contrasts, their transcript levels declined after 123 hpf, with the most pronounced reduction at 147 hpf. For *snad_810*, *snad_769*, *snad_448*, and *snad_946*, transcript abundance increased between 26 and 74 hpf and then remained relatively stable at later developmental stages, without major fluctuations (**Figures 1F, G–I**). In contrast, *snad_409*, *snad_208*, and *snad_962* remained at very low levels, close to zero, throughout embryogenesis and early larval development up to 123 hpf, after which transcript abundance increased rapidly and significantly (**Figures 1J–L**). Notably, *snad_318*, *snad_810*, and *snad_835* exhibited relatively high expression levels already at 0 hpf, whereas *snad_160*, *snad_448*, *snad_800*, *snad_063*, *snad_769*, and *snad_946* showed detectable transcript levels within the first hour post fertilization.

### 3.2 Hierarchical cluster analysis

**Figure 2:**
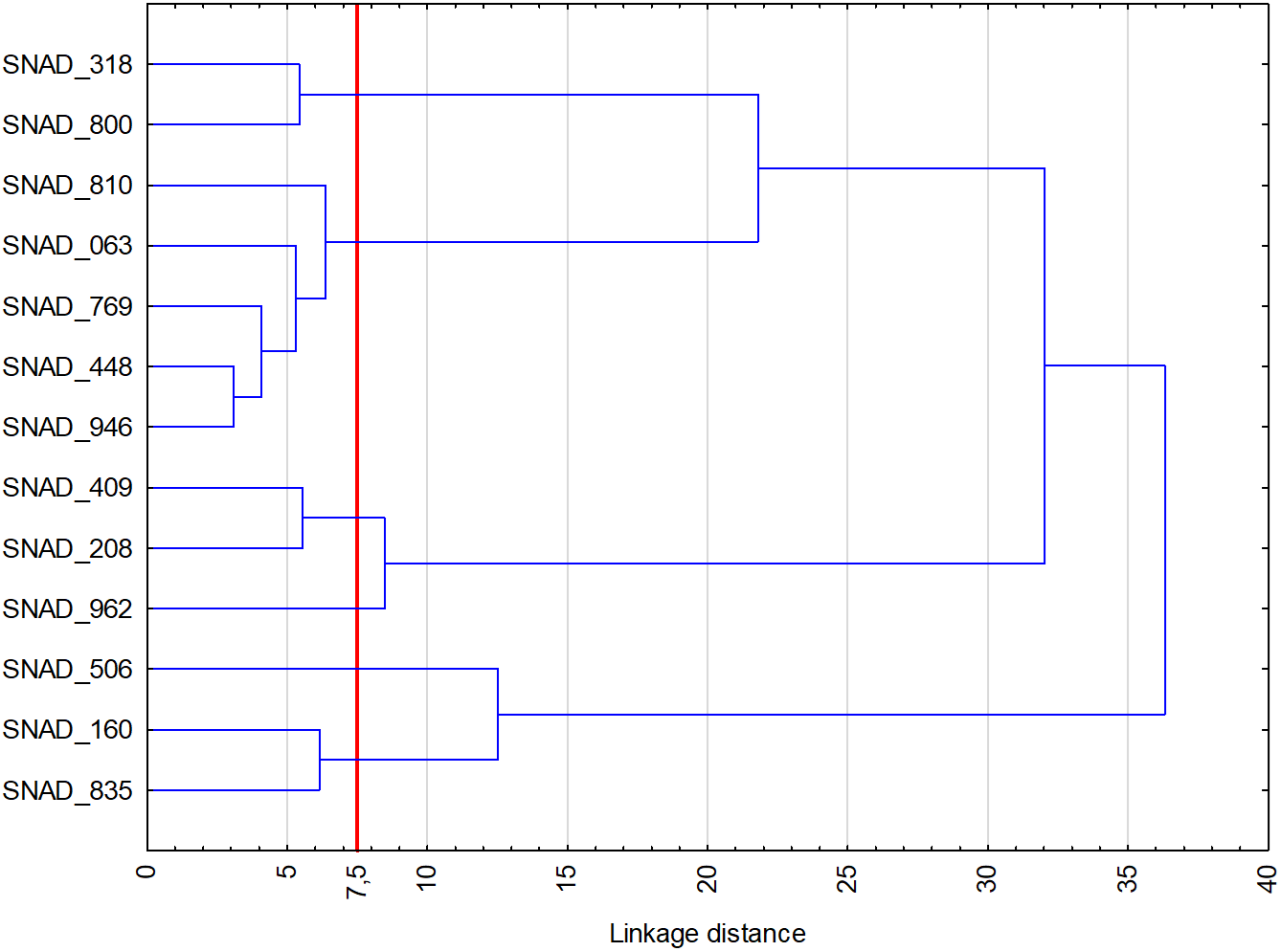
Hierarchical clustering of *snad1* gene expression profiles during common carp ontogeny. Dendrogram showing hierarchical relationships among *snad1* paralogs based on normalized expression values across developmental stages. The horizontal dashed line indicates the distance threshold (7.5) used to define six expression clusters.

We analyzed the expression profiles of all *snad1* genes using hierarchical clustering. The resulting dendrogram provided a graphical representation of the hierarchical relationships among the analyzed genes (**Figure 2**). Based on the clustering results, six distinct clusters were identified, each comprising genes with similar expression dynamics across different developmental stages of common carp. Cluster 1 contained only the *snad_506* gene, while Cluster 2 included exclusively *snad_962*. Clusters 3, 4, and 5 each comprised two *snad1* genes, specifically: *snad_318* and *snad_800* (Cluster 3), *snad_409* and *snad_208* (Cluster 4), and *snad_160* together with *snad_835* (Cluster 5). In contrast, Cluster 6 showed a close relationship among five *snad1* genes: *snad_810*, *snad_063*, *snad_769*, *snad_448*, and *snad_946*.

## 4 Discussion

This study provides the first insight into snad1 gene expression during the early ontogeny of common carp. We demonstrated that: (i) all 13 snad1 genes are expressed during carp ontogeny; (ii) 12 snad1 genes, excluding snad_506, exhibit significant stage-dependent variation in expression; and (iii) subsets of *snad1* paralogs were grouped into six distinct clusters based on their similar expression patterns across developmental stages, as revealed by hierarchical clustering.

### 4.1 Snad1s expression dynamics during carp ontogeny

The developmental expression profiles of 12 *snad1* paralogs reveal pronounced temporal heterogeneity during early ontogeny in common carp, suggesting functional diversification within this gene family, with four distinct temporal expression patterns corresponding to key developmental phases and closely associated with immunological and stress-related processes.

An exception among the analyzed genes was *snad_506*, which did not exhibit significant developmental variation in expression. Although its transcripts were consistently detected throughout ontogeny, expression remained relatively stable compared with the other *snad1* paralogs. The biological significance of this pattern remains unclear and may reflect functional divergence within the snad1 gene family.

Three *snad1* genes, including *snad_318*, *snad_835*, and *snad_810*, exhibited relatively high transcript abundance already at 0 hpf. Moreover, *snad_800*, *snad_063*, *snad_160*, *snad_769*, *snad_448*, *snad_946*, and *snad_506* showed detectable transcript levels within the first hour post fertilization, strongly suggesting a maternal origin of these transcripts. Expression detected prior to the onset of zygotic genome activation (ZGA) indicates maternal deposition, as early embryonic development is entirely dependent on maternally supplied biomolecules. In common carp maintained at 20 °C, ZGA occurs during the early blastula stage and spans approximately 2–3 hours, from ~3–5 to 7–8 hpf, after which embryonic development becomes predominantly governed by zygotic transcription (19). Before the maternal-to-zygotic transition, maternally deposited mRNAs serve as key regulators of early embryogenesis (7, 20). In contrast, *snad_409*, *snad_208*, and *snad_962* showed no detectable transcripts at fertilization but exhibited a marked increase only after ZGA, indicating a strictly zygotic origin.

**Figure 3:**
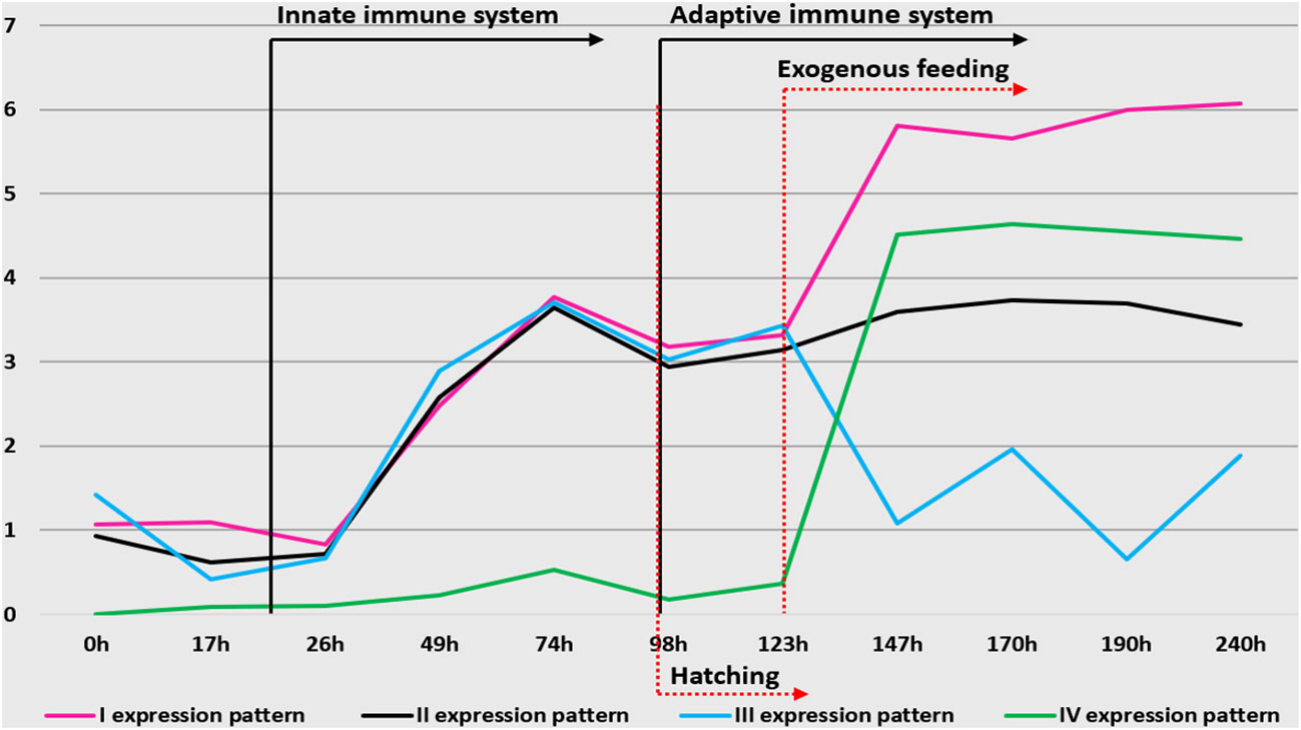
Major temporal expression patterns of *snad1* genes during common carp (*Cyprinus carpio*) development. Snad1 genes exhibiting highly similar temporal expression profiles were grouped, and average expression values were calculated to provide a schematic visualization of the major developmental expression trends observed during common carp ontogeny. **Figure 3** is intended as an illustrative summary of the expression patterns shown for individual genes in **Figure 1**. I expression pattern comprised (*snad_800, snad_318, snad_063*), II expression pattern comprised (*snad_810, snad_769, snad_448, snad_946*), III expression pattern comprised (*snad_835* and *snad_160*), IV expression pattern comprised (*snad_409, snad_208, snad_926*).

Based on these observations, four major temporal expression patterns were identified. To visualize these dynamics, average *snad1s* expression profiles were calculated, resulting in four representative patterns that reflect the main trends in *snad1* genes expression during development (**Figure 3**). Based on these observations, four major temporal expression patterns were identified. To visualize these dynamics, average snad1 expression profiles were calculated by averaging the expression values of genes exhibiting highly similar temporal expression trajectories. These profiles were intended to provide a simplified visualization of the major developmental trends rather than a formal statistical classification, resulting in four representative patterns that reflect the main trends in snad1 gene expression during development (**Figure 3**).

The first pattern comprised *snad_800*, *snad_318 and snad_063* and was characterized by an early post-embryonic upregulation followed by sustained expression during larval development. Previous studies have established the timing of major developmental events associated with immune system maturation in common carp, including the emergence of innate immune cell populations, thymus development, and the onset of adaptive immunity (11, 21, 22). Several *snad1* genes exhibited developmental activation during these same ontogenetic periods. The initial rise in expression at approximately 26 hpf coincided with late organogenesis and the onset of innate immune system activity. Around 48 hpf, the first cellular and humoral components of innate immunity emerge, including granulocytes and monocytes, together with detectable expression of major histocompatibility complex class II molecules (11, 21). Stabilization of expression between 74 and 123 hpf temporally overlapped with thymus development and the appearance of putative T cells and macrophages at approximately 74 hpf, marking the initiation of adaptive immune system formation (11, 22). Developmental events associated with immune-system formation continue during this period, coinciding with hatching and subsequent larval development. During this period, expression of the above-mentioned *snad1* genes remained relatively stable and persisted until the transition from yolk-based nutrition to exogenous feeding at approximately 123 hpf. This transition was followed by a marked increase in expression, indicating developmental regulation during early larval life.

A closely related second expression pattern was observed for *snad_810*, *snad_769*, *snad_448*, and *snad_946*. These genes exhibited a comparable early increase in expression during the initial stages of development; however, after 74 hpf their transcript levels remained relatively stable, without a pronounced secondary upregulation. This sustained profile suggests continuous transcriptional demand.

The third temporal expression pattern was represented by *snad_835* and *snad_160*, which exhibited an early expression profile similar to the first pattern but differed in showing a pronounced decline in transcript levels by 123 hpf. This biphasic expression pattern suggests involvement in early immune-related developmental processes, followed by reduced activity during later larval stages, potentially reflecting functional specialization during early ontogeny.

The fourth temporal expression pattern was observed for *snad_409*, *snad_208*, and *snad_926*, which displayed a distinctly delayed onset of expression. Transcript levels for these genes remained very low throughout embryogenesis and early larval development, with a rapid increase only after 123 hpf. This expression profile coincides with post-hatching maturation of immune-related physiological processes and the establishment of host defense mechanisms during early larval development.

Taken together, the identification of four distinct temporal expression patterns, divided into early induction with sustained activation, early induction followed by repression, and late-induction, highlights the functional heterogeneity of the *snad1* gene family during common carp ontogeny. The close temporal association between snad1 gene activation and major developmental transitions, including periods associated with immune-system formation, identifies these genes as candidates for future functional investigations.

Nevertheless, the present study aimed at characterizing developmental expression dynamics rather than establishing functional relationships between Snad1 proteins and immune system development. Therefore, the proposed biological interpretations should be considered as hypotheses requiring experimental validation through future localization and gene-manipulation studies.

### 4.2 Cluster-based analysis of expression changes in snad1 genes across developmental stages

Hierarchical clustering analysis revealed substantial heterogeneity in the developmental expression dynamics of *snad1* paralogs and identified six distinct expression clusters. The observed grouping of genes with similar temporal expression profiles suggests coordinated transcriptional regulation among selected *snad1* paralogs during common carp ontogeny.

Notably, *snad_506* and *snad_962* formed independent single-gene clusters, indicating expression dynamics distinct from those of the remaining paralogs. In contrast, the multi-gene clusters comprised paralogs with highly similar developmental expression profiles, suggesting partially conserved regulatory mechanisms and potential functional relationships.

Overall, these findings indicate substantial heterogeneity in the developmental expression dynamics of *snad1* paralogs while revealing subsets of genes with conserved temporal expression patterns during early ontogeny. Furthermore, coordinated regulation of specific snad1 paralogs coincides with developmental periods associated with the establishment of immune competence during early life stages.

### 4.3 Common carp snad1s in RNA-seq datasets

Currently, information regarding *snad1* gene expression during fish embryogenesis remains very limited. Therefore, to further validate our observations, we reanalyzed publicly available RNA-seq data generated by Luo et al. (23). In this study, embryonic and early larval development of koi carp (*Cyprinus carpio* var. koi) was investigated at the following developmental stages: multicellular (0.2–2.3 hpf), blastula (3.3–5.0 hpf), gastrulation (5.0–8.0 hpf), neurula (8.0–9.15 hpf), organogenesis (16.0–23 hpf), and 7 days post-hatching (168 hpf). Reanalysis of these raw RNA-seq datasets provided independent confirmation that all thirteen *snad1* genes are expressed during embryonic and early larval development. Owing to differences in developmental sampling design and analytical methodology, the comparison was intended to assess transcript presence and overall developmental expression rather than to perform direct quantitative comparisons between datasets.

It should be emphasized, however, that the developmental stages analyzed by Luo et al. differed from those examined in the present study, and the datasets were generated using RNA-seq rather than multiplex PCR. These methodological and sampling differences limit direct quantitative comparisons between studies. Nevertheless, the concordance between the independently generated datasets strongly supports the temporal expression of *snad1* genes during early embryogenesis and further suggests that *snad1*-related genes may play evolutionarily conserved roles in embryonic development and immune system maturation across carp lineages.

## 5 Concluding remarks

This study provides the first comprehensive characterization of the expression dynamics of all 13 *snad1* genes during early ontogeny of the common carp (*Cyprinus carpio*). The obtained results demonstrate that *snad1* paralogs are transcriptionally active from the earliest developmental stages and display distinct temporal expression profiles. Several *snad1* genes are already expressed at fertilization, suggesting a maternal origin and potential involvement in early embryogenesis, whereas others are activated later, coinciding with the development of innate and adaptive immune functions and the transition to exogenous feeding.

Our analyses identified four major temporal expression patterns, including early induction with sustained activation, early induction followed by repression, and late induction, as well as six distinct hierarchical expression clusters, highlighting substantial functional heterogeneity within the *snad1* gene family. At the same time, the observed co-expression of selected paralogs suggests the existence of partially conserved regulatory mechanisms operating during common carp ontogeny. Notably, the temporal distribution of these expression profiles closely parallels key developmental transitions, including periods associated with immune-system formation during early life stages. Comparison with publicly available RNA-seq datasets from koi carp further confirmed the conserved developmental expression of *snad1* genes across *Cyprinus* lineages, reinforcing the hypothesis that members of the SNAD1 family play evolutionarily conserved roles during early vertebrate development.

Taken together, our findings suggest that SNAD1 proteins represent a teleost-specific expansion of the AID/APOBEC-like cytidine deaminase family with potentially diversified biological functions. The distinct temporal expression trajectories observed among individual *snad1* paralogs indicate functional diversification during ontogeny, whereas their coordinated activation during periods of immune system maturation points to a potential role in the development of early immune competence. Future studies should focus on identifying the molecular targets, regulatory pathways, and functional specialization of individual *snad1* paralogs to better understand their contribution to fish immunity, embryonic development, and environmental adaptation.

## Data Availability

The original contributions presented in the study are included in the article/**Supplementary Material**. Further inquiries can be directed to the corresponding author.
