# Supplementary material for: Developmental expression of immune-associated secreted novel AID/APOBEC-like deaminases (SNAD1s) in common carp (Cyprinus carpio)
Source: Front Immunol. 2026 Jul 13;17:1893787. doi: 10.3389/fimmu.2026.1893787 (PMC13402138; doi:10.3389/fimmu.2026.1893787)
Supplement: Supplementary file 1 [file Table1.docx]

**Supplementary Table1.** Summary of the embryonic and early larval development of common carp (*Cyprinus carpio*). Data include sampling times, developmental forms, and corresponding measurements of water temperature during the observation period.

| **Number** | **Date** | **Time** | **Hours** | **Form** | **Water temperature** |
| --- | --- | --- | --- | --- | --- |
| I | 15.05.2024 | 10:00 | (T -1) | eggs | - |
| II | 15.05.2024 | 11:00 | 0 (T0) | eggs | 18°C |
| III | 16.05.2024 | 04:00 | 17 h | eggs | 19.4°C |
| IV | 16.05.2024 | 13:00 | 26h (1 day) | eggs | 19.1°C |
| VI | 17.05.2024 | 12:00 | 49h (2 days) | eggs | 19°C |
| VII | 18.05.2024 | 13:00 | 74h (3 days) | eggs | 19°C |
| VIII | 19.05.2024 | 13:00 | 98h (4 days; hatching the fry 20:30) | fry | 20°C |
| IX | 20.05.2024 | 14:00 | 123h (5 days) | fry | 21.4°C |
| X | 21.05.2024 | 14:00 | 147h (6 days) | fry | 20.1°C |
| XI | 22.05.2024 | 13:00 | 170h (7 days) | fry | 18.6°C |
| XII | 23.05.2024 | 09:00 | 190h (8 days) | fry | 17.5°C |
