# Supplementary material for: Developmental expression of immune-associated secreted novel AID/APOBEC-like deaminases (SNAD1s) in common carp (Cyprinus carpio)
Source: Front Immunol. 2026 Jul 13;17:1893787. doi: 10.3389/fimmu.2026.1893787 (PMC13402138; doi:10.3389/fimmu.2026.1893787)
Supplement: Supplementary file 2 [file Table2.docx]

| **Gene** | **Primer forward (5ʹ- 3ʹ)** | **Primer reverse (5ʹ- 3ʹ)** | **Amplicon length [bp]** |
| --- | --- | --- | --- |
| *LOC109045318* | GACAATGTCCAGCCAAAGACCG | AGGATGATGCAGCACAAGTGAAA | 138 |
| *LOC109051800* | GGAGTCAGTAGCCATCAATGTC | TGTGAGAAATATCATACGTGCTGA | 120 |
| *LOC109060409* | ATCTACGGGGTCTCGTCATCG | GGTGAACACTGTGTGTCTTGCT | 87 |
| *LOC109068208* | CGAGCAAAACTACAAGAGAGTTG | TCTCAAGAGAATGCCATGAATGTG | 148 |
| *LOC109070810* | CACAATGGCGTCTGGGGTACT | GCATTGAGGGTGTGAACACTGT | 93 |
| *LOC109096506* | CAGCAGTCACAATGGCACCAC | CGACGTGAACACTGCCGCTCT | 97 |
| *LOC109104160* | CTACTGCTATAAGAGAGTTGATGA | AAGAACTTCAGTCCTGCACAGAA | 92 |
| *LOC109107063* | CATCGAAGTTATGGAAGCAGTAGT | ACCTTACATAAAACGTGACAGCAA | 152 |
| *LOC109107769* | TGACCATGTCCAGCCAACCAG | AGCAAAAAGGATGCAGAAAATGTG | 130 |
| *LOC122138962* | TTGCTGACAATGTCCAGCCAAAA | TAGATGCTGATATCCAGAATGTGT | 114 |
| *LOC122140448* | TGGTTTTCACTAAGCTGTCAAGG | GAGTTCGACGTGGAAGTTCTTG | 96 |
| *LOC122141946* | TGCAGAGCGTCTCTTACTCACTT | TATCTTCTACAGCTACAAATCTCC | 114 |
| *LOC122147835* | ATGTCCAGCCAAAGACCAAGATA | CATATTGAGAATGTGTTCAGCAGG | 113 |

**Supplementary Table 2.** List of primers used in this study.
